# IGFBP2 plays an important role in heat shock protein 27-mediated cancer progression and metastasis

**DOI:** 10.18632/oncotarget.18989

**Published:** 2017-07-05

**Authors:** Chin-Sheng Hung, Chien-Yu Huang, Chia-Hwa Lee, Wei-Yu Chen, Ming-Te Huang, Po-Li Wei, Yu-Jia Chang

**Affiliations:** ^1^ Graduate Institute of Clinical Medicine, College of Medicine, Taipei Medical University, Taipei, Taiwan, ROC; ^2^ Department of Surgery, School of Medicine, College of Medicine, Taipei Medical University, Taipei, Taiwan, ROC; ^3^ Division of General Surgery, Department of Surgery, Taipei Medical University Hospital, Taipei, Taiwan, ROC; ^4^ Division of General Surgery, Department of Surgery, Shuang Ho Hospital, Taipei Medical University, Taipei, Taiwan, ROC; ^5^ School of Medical Laboratory Science and Biotechnology, College of Medical Science and Technology, Taipei Medical University, Taipei, Taiwan, ROC; ^6^ Department of Pathology, School of Medicine, College of Medicine, Taipei Medical University, Taipei, Taiwan, ROC; ^7^ Department of Pathology, Wan Fang Hospital, Taipei Medical University, Taipei, Taiwan, ROC; ^8^ Division of Colorectal Surgery, Department of Surgery, Taipei Medical University Hospital, Taipei Medical University, Taipei, Taiwan, ROC; ^9^ Translational Laboratory, Department of Medical Research, Taipei Medical University Hospital, Taipei Medical University, Taipei, Taiwan, ROC; ^10^ Graduate Institute of Cancer Biology and Drug Discovery, Taipei Medical University, Taipei, Taiwan, ROC; ^11^ Cancer Research Center, Taipei Medical University Hospital, Taipei, Taiwan, ROC

**Keywords:** HCC, Hsp27, IGFBP2

## Abstract

Heat shock protein 27 (Hsp27) is a key chaperone that interacts with over 200 client proteins. The expression of Hsp27 might be correlated with poor outcome in many types of cancer. Previous study indicated that Hsp27 might be an important biomarker in hepatocellular carcinoma (HCC). However, the detailed mechanism is less well understood. The shRNA-mediated silencing of Hsp27 decreased the proliferation, migration and invasion of HCC cells. In a xenograft model, the silencing of Hsp27 reduced tumor progression. We revealed that the silencing of Hsp27 led to a reduction in insulin-like growth factor binding protein 2 (IGFBP2), which might mediate proliferation and metastasis through vimentin, snail and beta-catenin. The overexpression of IGFBP2 reversed the reductions in cell growth, migration and invasion. The tissue array results showed that HCC patients with high Hsp27 expression exhibited poor prognosis and increased metastasis. The Hsp27 expression was highly correlated with IGFPB2 in CRC specimen. ChIP and luciferase assays showed that Hsp27 does not directly bind the IGFBP2 promoter region to regulate the transcription of IGFBP2. In conclusion, our study demonstrated that Hsp27 is a key mediator of HCC progression and metastasis and that Hsp27 might regulate proliferation and metastasis through IGFBP2. This pathway might provide a new direction for the development of a novel therapeutic strategy for HCC.

## INTRODUCTION

Cancer is a major cause of morbidity and mortality worldwide, with 8.2 million cancer-related deaths in 2012. Hepatocellular carcinoma (HCC) is the second most common cause of cancer-related death in the world, and approximately half of these deaths occur in China and Southeast Asia [1]. HCC is associated with a poor outcome because less than 20% of HCC patients can undergo surgery for complete resection; the remaining patients receive transcatheter arterial chemoembolization, radiofrequency ablation, chemotherapy or targeted therapy, all of which have higher recurrence rates than surgery. Despite progress in developing anti-cancer therapeutics, the outcome of HCC is still poor, and HCC usually causes death within 3 to 6 months in cases of unresectable disease [2, 3]. Therefore, it is important to determine how to treat, prevent, and detect HCC.

Infectious agents induce inflammation and are directly implicated in tumorigenesis, which involves both the innate and adaptive components of the immune system, via regulation of tumor angiogenesis, tumor tolerance and metastatic behavior [4–6]. Hepatitis B virus and hepatitis C virus are the most important risk factors for HCC. Although early vaccination against hepatitis B virus can effectively prevent HCC [7–9], the inhibition of tumorigenesis after infection remains an important hurdle for the further control or prevention of HCC.

The production of high levels of heat shock proteins can be triggered by inflammation, and this upregulation is part of stress responses [10, 11]. These proteins provide a link between infection-mediated inflammation and subsequent cancer development [4, 12]. The expression level of some heat shock proteins, such as heat shock protein 27 (Hsp27) and heat shock protein 70 (Hsp70) in particular, can determine cell fate in response to a death stimulus [13]. There is evidence that Hsp27, Hsp70 and Hsp90 are powerful anti-apoptotic proteins with the capacity to block the cell death process at different levels [14]. Those studies imply that Hsp27 plays an important role in regulating apoptosis and might participate in carcinogenesis.

Insulin-like growth factors (IGFs) play an important role in many diseases, particularly cancer and diabetes [15, 16]. Some researchers recently identified a family of proteins that regulate IGFs, namely the IGF-binding proteins (IGFBPs). These proteins modulate the functions of IGFs and might regulate cancer metastasis- and invasion-associated signaling networks [17]. In HCC, the circulating IGF and IGFBP levels are strongly associated with liver status [18]. IGFBP2, a key member of the IGF family, has been reported as a notable oncogene in most human epithelial cancers [17, 19]. However, the factors that regulate IGFBP2 and the relationship between IGFBP2 and heat shock proteins remain unknown.

In our study, we found that Hsp27 plays an essential role in HCC progression and metastasis and that the Hsp27 levels reflect the HCC patient outcome. Mechanistically, we found that Hsp27 might regulate proliferation and metastasis through IGFBP2.

## RESULTS

### Hsp27 is highly expressed in HCC cells

**Figure 1:**
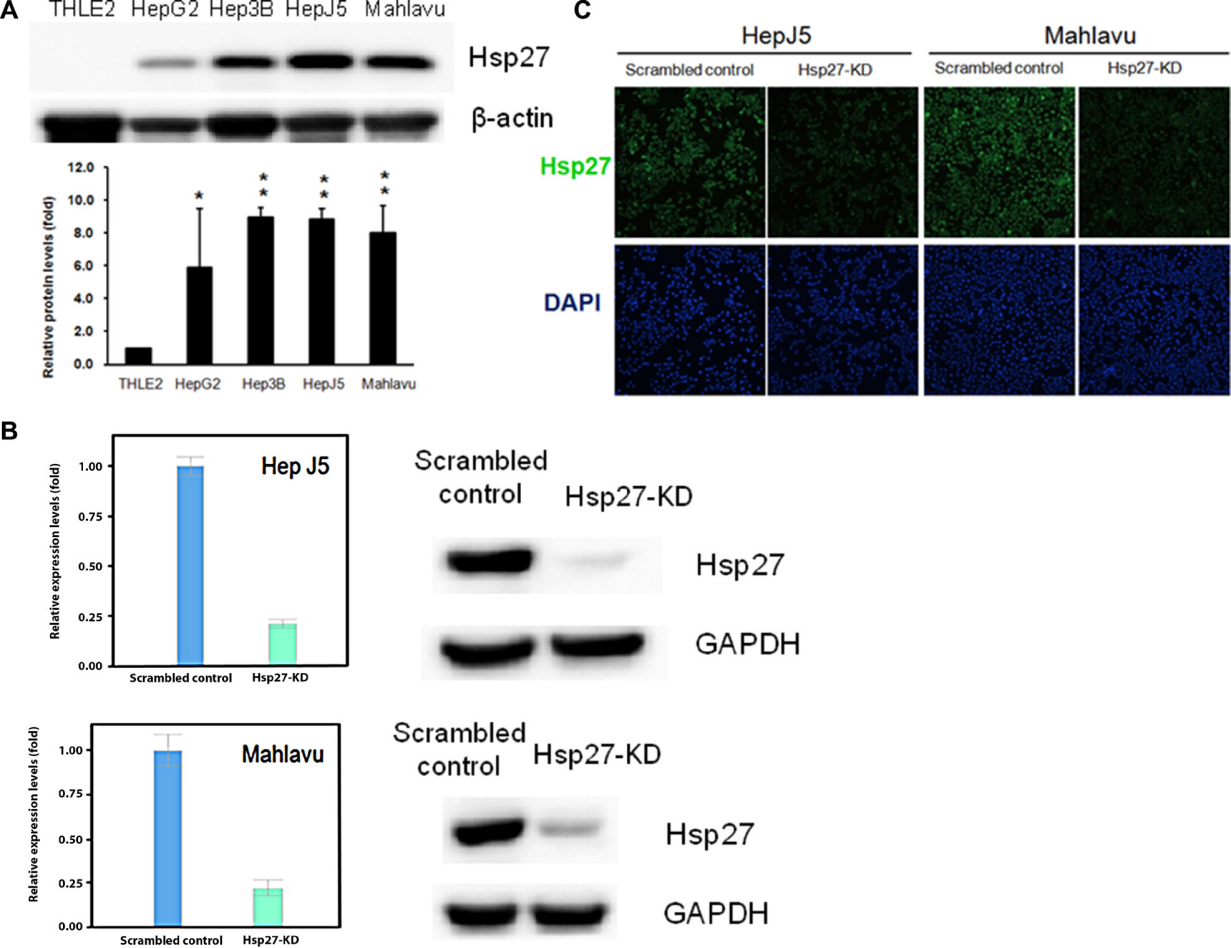
The expression level of Hsp27 (**A**) The levels of Hsp27 in normal liver cell and HCC cell lines were determined by western blotting. (**B**) Generation of Hsp27-knockdown (Hsp27-KD) HCC cells. Hsp27 expression in scrambled control and Hsp27-KD HepJ5 or Mahlavu cells was determined by western blotting. (**C**) Hsp27 expression in scrambled control and Hsp27-KD cells was determined by immunofluorescent staining.

First, we determined Hsp27 expression levels in normal liver cells (THLE2) and HCC cells (HepG2, Hep3B, Hep-J5, and Mahlavu) by western blotting. Hsp27 was more highly expressed in HCC cells compared with THLE2 cells (Figure 1A). We silenced Hsp27 expression in Hep-J5 and Mahlavu cells using shRNA, and stably transfected cells were selected with puromycin. The Hsp27 levels were determined by quantitative PCR and western blotting (Figure 1B). Immunofluorescence staining revealed markedly reduced fluorescence intensity in Hsp27 knockdown (Hsp27-KD) cells (Figure 1C).

### Role of Hsp27 in cancer cell proliferation

**Figure 2:**
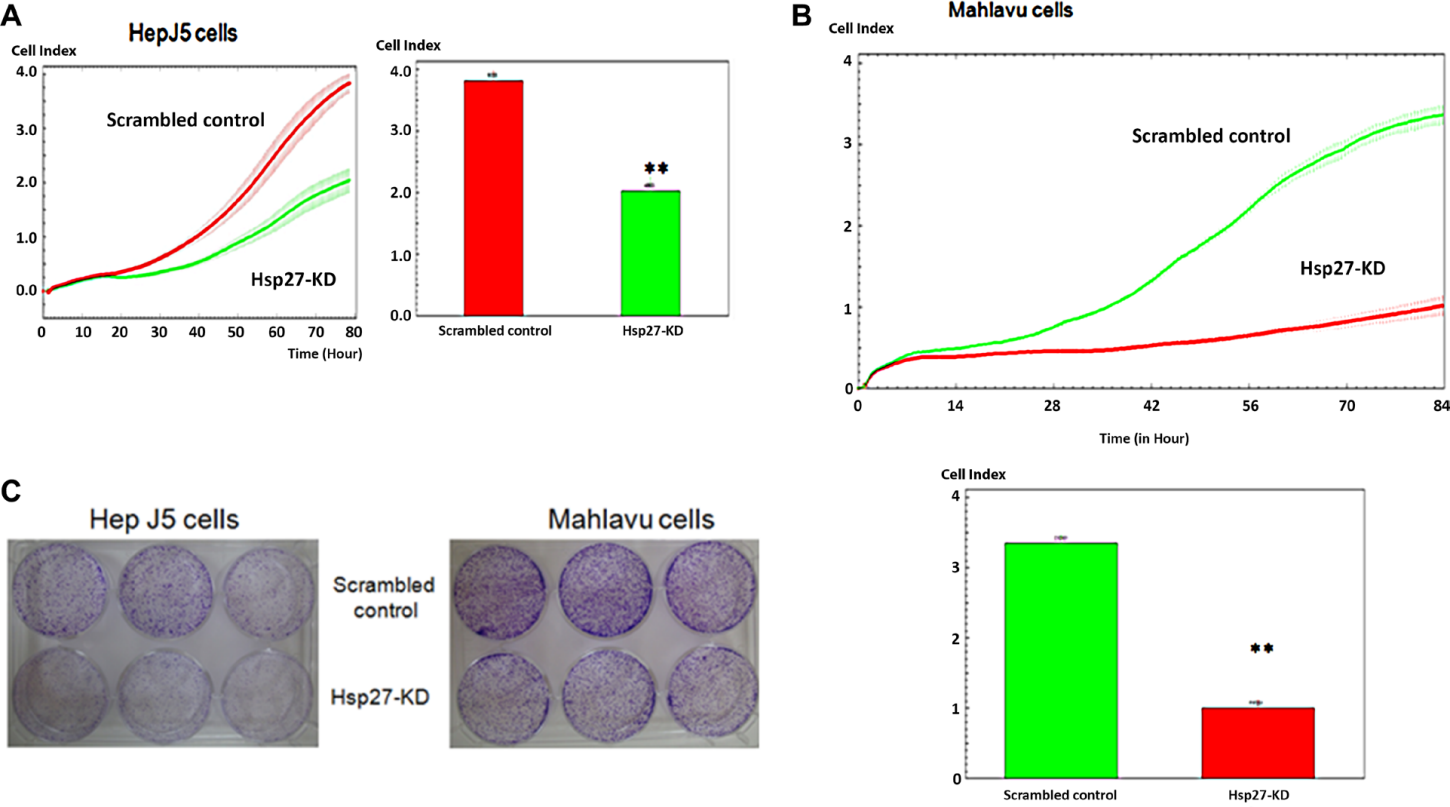
Hsp27 mediates the growth activity of HCC cells The proliferation activity of scrambled control and Hsp27-KD HCC cells was determined by x-CELLigence biosensors in (**A**) HepJ5 and (**B**) Mahlavu cells. (**C**) Colony formation was assayed in scrambled control and Hsp27-KD HCC cells. All experiments were repeated at least three times independently. **indicates *p* < 0.01.

We performed proliferation assays using the real-time x-CELLigence biosensor system to ascertain the role of Hsp27 in HCC progression. The silencing of Hsp27 markedly reduced the proliferation of HepJ5 and Mahlavu cells (Figure 2A–2B), indicating that Hsp27 plays a key role in HCC growth. In addition, we performed colony formation assays to evaluate cell stemness. As shown in Figure 2C, there were more colonies in the scrambled control group than in the Hsp27-KD group. These results suggest that Hsp27 plays a major role in the progression of HCC.

### Silencing Hsp27 reduces HCC xenograft formation and growth in an animal model

**Figure 3:**
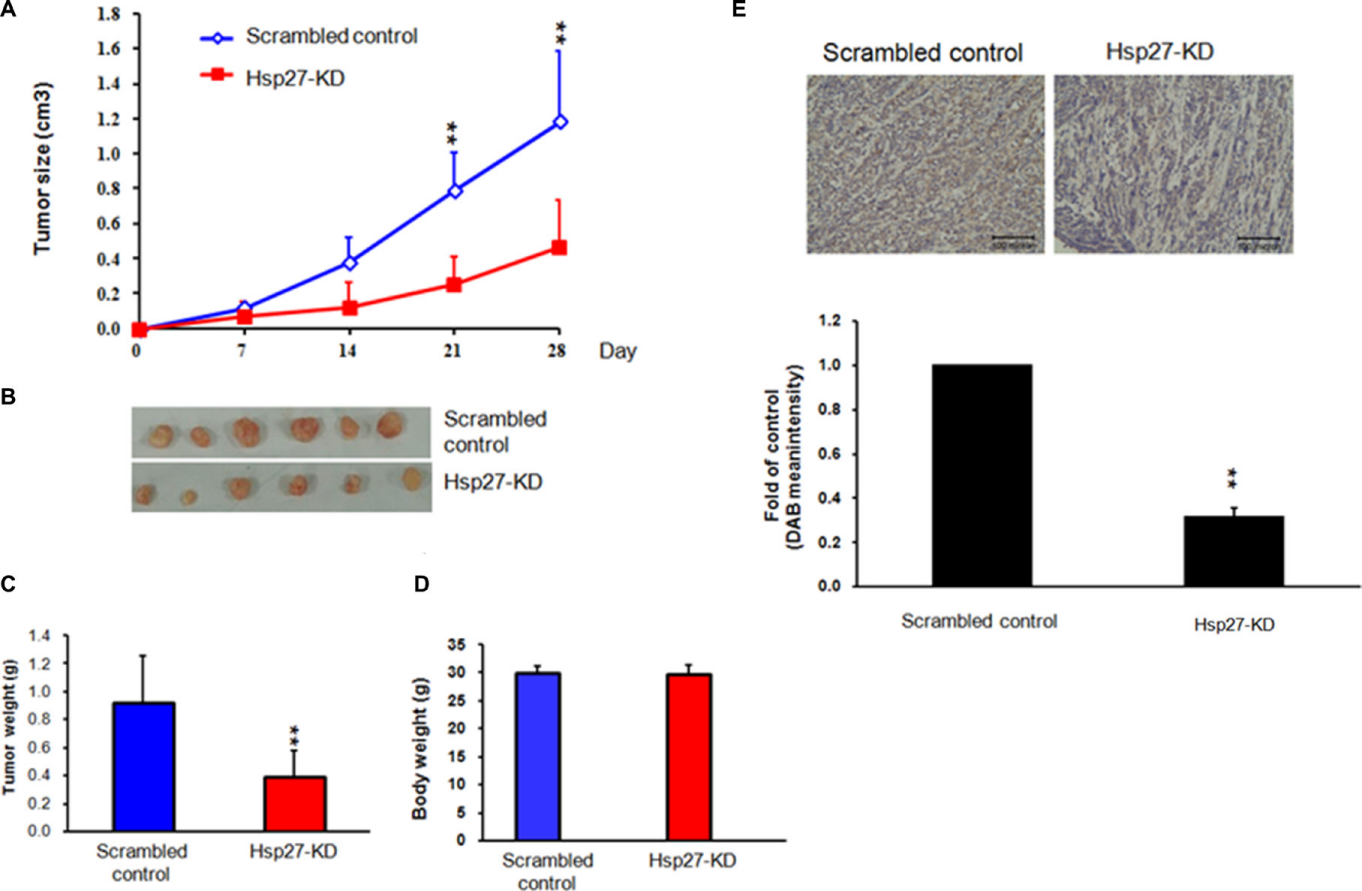
Hsp27 mediates cancer progression in a xenograft model (**A**) Scrambled control and Hsp27-KD HepJ5 cells were inoculated subcutaneously into nude mice. The sizes of the tumors were monitored weekly, and the animals were sacrificed at day 28. (**B**–**C**) The weights of Hsp27-KD tumors were markedly lower than those of scrambled tumors. (**D**) The body weights of the scrambled control group and Hsp-27KD group were similar. (**E**) IHC staining indicated decreased Hsp27 staining in Hsp27-KD tumors.

To further determine the role of Hsp27 in cancer progression, we implanted scrambled control and Hsp27-KD Hep-J5 cells into nude mice. The tumor volume was measured once per week. As shown in Figure 3A, the growth curve for the scrambled control group was higher than that for the Hsp27-KD group. The mice were sacrificed at day 28 after implantation. The tumors were smaller in the Hsp27-KD group compared with the scrambled control group (Figure 3B–3C). There was no effect on body weight (Figure 3D). Immunohistochemical staining confirmed that Hsp27 expression was lower in Hsp27-KD tumors than in control tumors (Figure 3E). The *in vivo* xenograft results indicated that Hsp27 might mediate HCC progression.

### Silencing Hsp27 influences the migratory and invasive abilities of HCC cells

**Figure 4:**
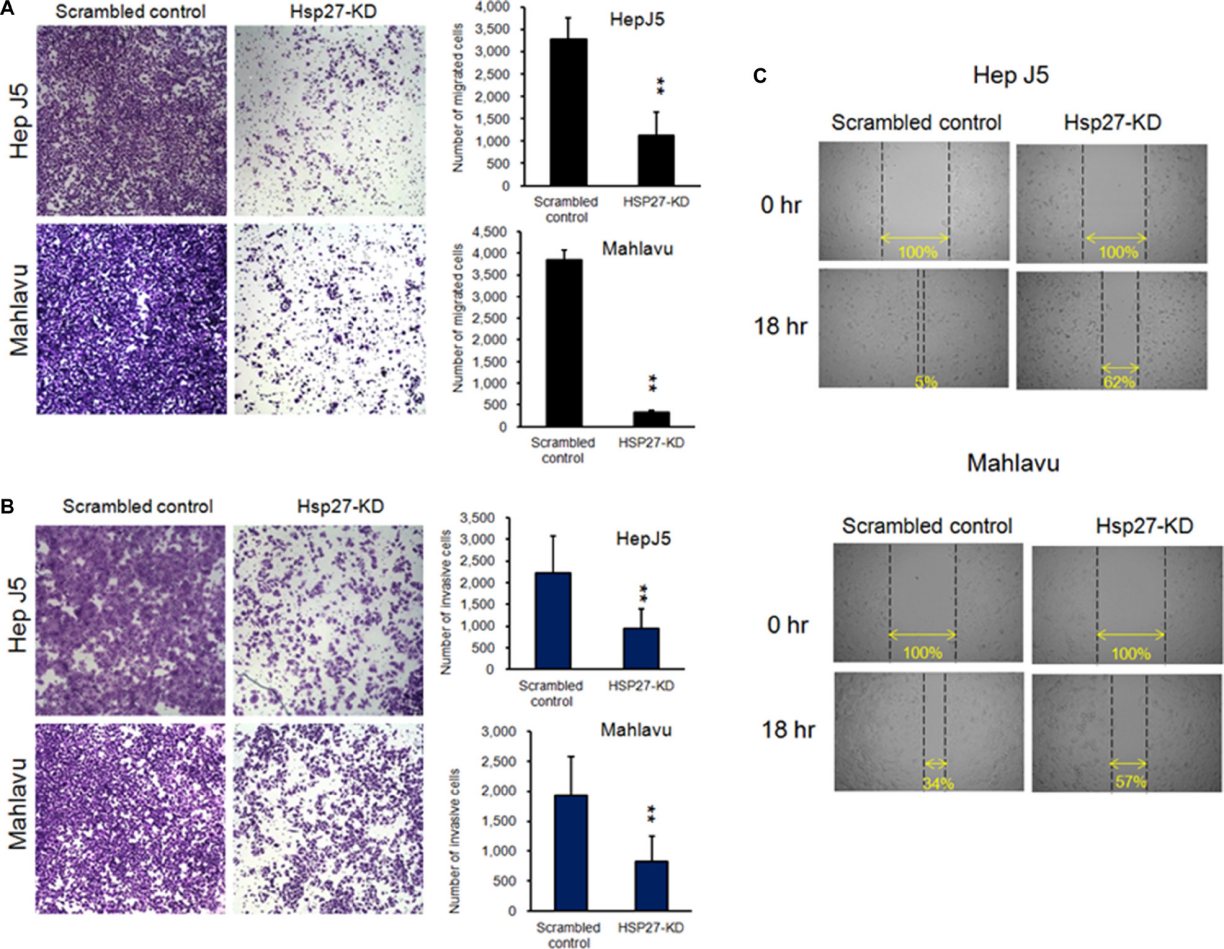
Silencing Hsp27 decreases HCC cell metastasis HepJ5 and Mahlavu Hsp27KD cells exhibited lower migratory and invasive abilities (**A**), (**B**). HepJ5 and Mahlavu Hsp27KD cells also exhibited poor wound healing ability. (**C**) The wound-healing migration assay was performed using the ibidi system. The migratory abilities of scrambled control and Hsp27-KD cells were determined based on the area of the wound gap.

Cell mobility is a key indicator of malignant tumor progression. Metastasis is also an important issue in clinical therapeutics. We further analyzed the role of Hsp27 in HCC cell migration using the x-CELLigence biosensor system and Transwell migration assays. As shown in Figure 4A, the migratory behavior in the Transwell migration assay was reduced more than 50% after the knock down of Hsp27 in both Hep-J5 and Mahlavu cells. The invasion assay revealed markedly fewer invasive Hsp27-KD cells than invasive scrambled control cells (Figure 4B). The wound healing ability of Hsp27-KD cells was reduced compared with that of scrambled control cells in the Hep-J5 and Mahlavu cell (Figure 4C). These results reveal that Hsp27 mediates the migratory ability of HCC cells.

### Clinicopathological significance of Hsp27 expression in HCC

**Figure 5:**
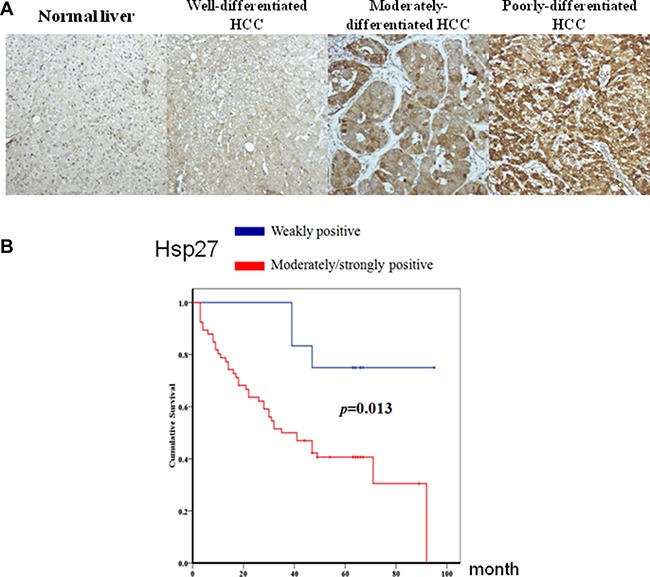
Associations between clinicopathological features and Hsp27 expression in hepatocellular carcinoma (HCC) (**A**) Hsp27 expression was stronger in HCC than in normal liver tissue. Poorly and moderately differentiated HCC expressed higher Hsp27 levels compared with well-differentiated HCC (*p* = 0.002). (**B**) Moderate/strong Hsp27 expression was associated with poorer prognosis in HCC patients (*p* = 0.013).

To better understand the prognostic role of Hsp27 in HCC, Hsp27 immunohistochemistry was performed using HCC tissue microarrays. The associations between clinicopathological characteristics and Hsp27 expression are shown in Table 1. Hsp27 expression was stronger in HCC than in normal liver tissue (Figure 5A). Higher-grade HCC tended to show stronger expression of Hsp27 (*p* = 0.002) (Figure 5A). Hsp27 expression was not correlated with patient age, gender or stage. A Kaplan-Meier curve analysis showed that the overall survival of patients with weak Hsp27 expression was significantly better than that of patients with moderate/strong Hsp27 expression (*p* = 0.013) (Figure 5B). Our previous study showed that higher Hsp27 expression correlated with increased HCC metastasis [20]. These results indicate that Hsp27 plays a key role in the progression and metastasis of HCC.

**Table 1:** Associations between the clinicopathological characteristics of hepatocellular carcinoma and HSP27 expression

Characteristics	HSP27 expression			*p* value
	Weak (1+)	Moderate (2+)	Strong (3+)	
**Gender**				0.456
Male	8 (13%)	32 (52%)	21 (35%)	
Female	4 (21%)	7 (37%)	8 (42%)	
**Age**				0.890
< 65 years	10 (16%)	30 (48%)	23 (36%)	
≧65 years	2 (12%)	9 (53%)	6 (35%)	
**Grading of HCC**				0.002*
Well-differentiated	6 (35%)	10 (59%)	1 (6%)	
Moderately differentiated	6 (14%)	22 (50%)	16 (36%)	
Poorly differentiated	0 (0%)	7 (37%)	12 (63%)	
**T status (TNM staging system; AJCC 7th Ed)**				0.661
T1 + T2	8 (17%)	21 (45%)	18 (38%)	
T3 + T4	4 (12%)	18 (55%)	11 (33%)	
**Stage (AJCC 7th Ed)**				0.661
I + II	8 (17%)	21 (45%)	18 (38%)	
III + IV	4 (12%)	18 (55%)	11 (33%)	

Abbreviations: HCC, hepatocellular carcinoma; AJCC, American Joint Committee on Cancer; TNM, Tumor size-lymph node-metastasis.

### Silencing Hsp27 influences EMT biomarkers

**Figure 6:**
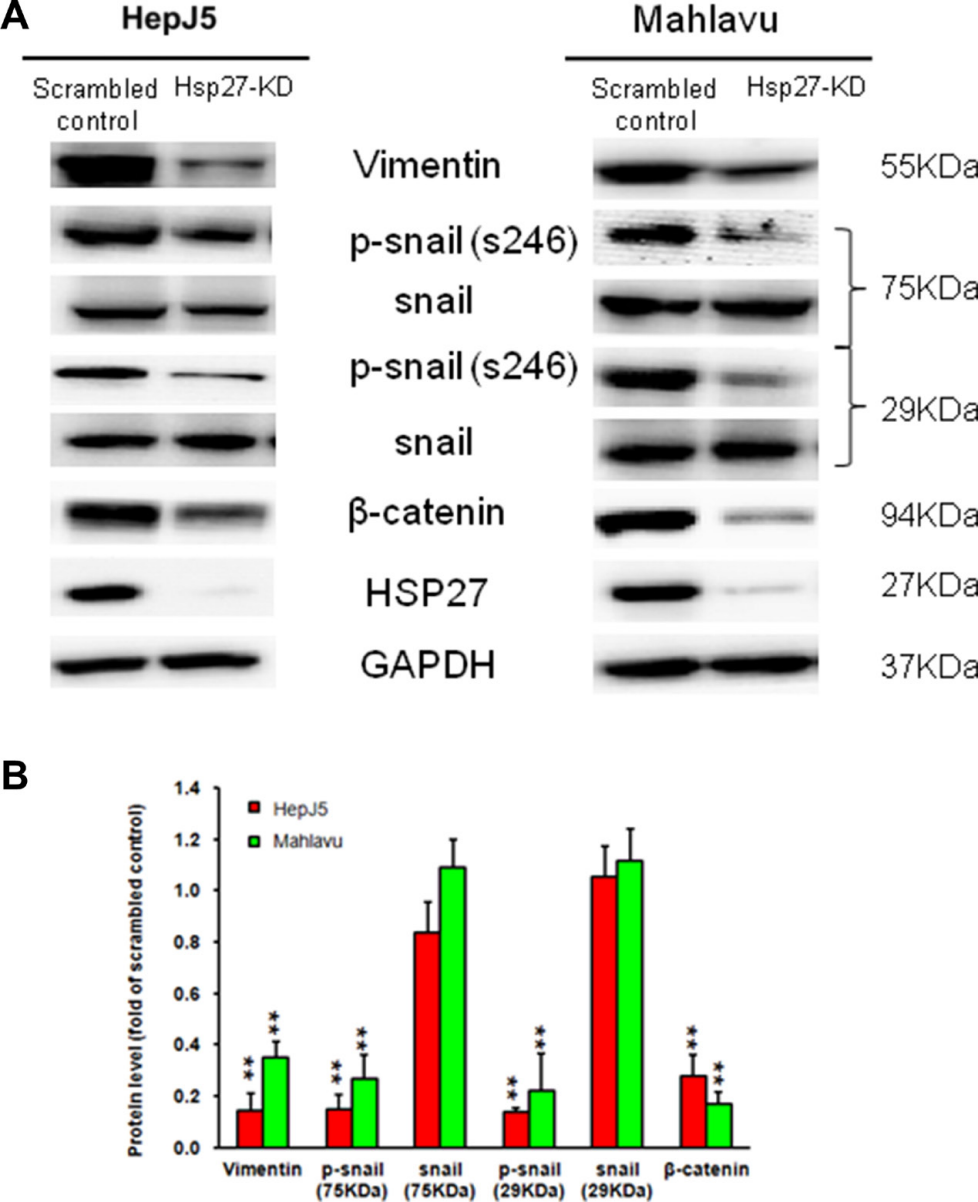
Hsp27 regulates EMT marker expression in HCC The silencing of Hsp27 downregulated mesenchymal cell markers in HepJ5 and Mahlavu cells. GAPDH was used as an internal control. **p < 0.01.

We further ascertained the possible mechanism through which Hsp27 mediates HCC metastasis. We checked the levels of vimentin, snail, phospho-snail, and β-catenin. As shown in Figure 6, we found decreased vimentin, phospho-snail, and β-catenin levels, but there was no change in snail expression. These results suggest that the silencing of Hsp27 in HCC cells might reduce metastasis by affecting the vimentin, phospho-snail, and β-catenin levels.

### IGFBP2 is a downstream gene of Hsp27 that modulates HCC progression

**Figure 7:**
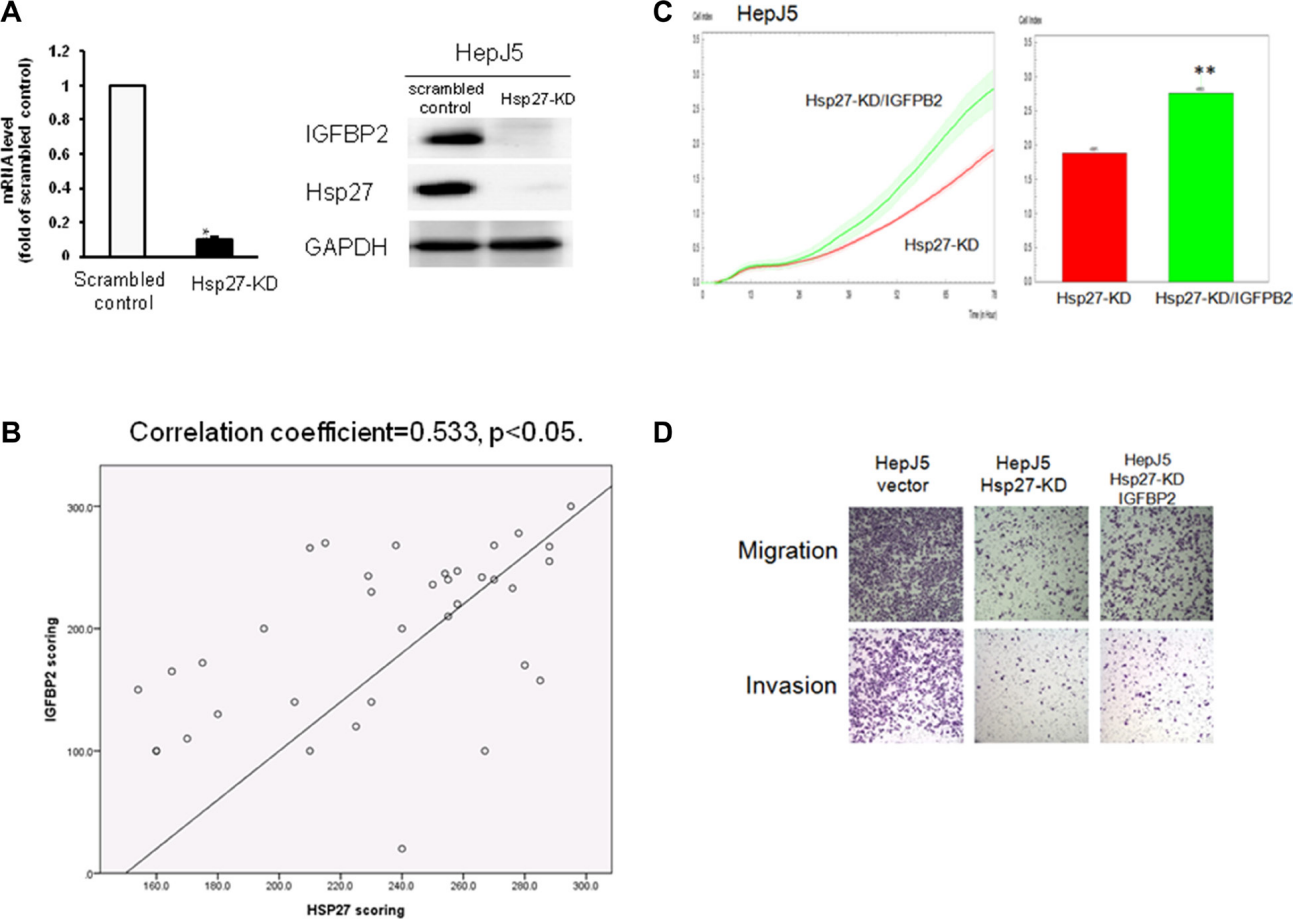
IGFBP2 is a downstream gene of Hsp27 The silencing of Hsp27 decreased the IGFBP2 (**A**) mRNA and protein levels in HepJ5 cells. (**B**) The expression correlation of Hsp27 and IGFBP2 was found in tissue array. The overexpression of IGFBP2 partially reversed the deficits in proliferation (**C**), migration and invasion (**D**) in Hsp27-KD HepJ5 cells.

We performed real-time PCR to determine the IGFBP2 expression levels. As shown in Figure 7A, the IGFBP2 levels were downregulated in Hsp27-KD cells, and we confirmed this result by western blotting (Figure 7A). We also checked the correlation between Hsp27 and IGFBP2 through a tissue array. As shown in Figure 7B, the correlation coefficient was 0.533, indicating that the Hsp27 levels were correlated with IGFBP2 expression in HCC specimens. We further overexpressed IGFBP2 in Hsp27-KD cells and performed proliferation, migration and invasion assays. As shown in Figure 7C–7D, the overexpression of IGFBP2 in Hsp27-KD cells increased cell growth, migration, and invasion but did not completely reverse the effects of silencing Hsp27. These results suggest that IGFBP2 is a downstream gene of Hsp27 and mediates cell proliferation, migration and invasion.

### Overexpressing IGFBP2 reverses the effects of Hsp27 on metastasis via EMT biomarkers

**Figure 8:**
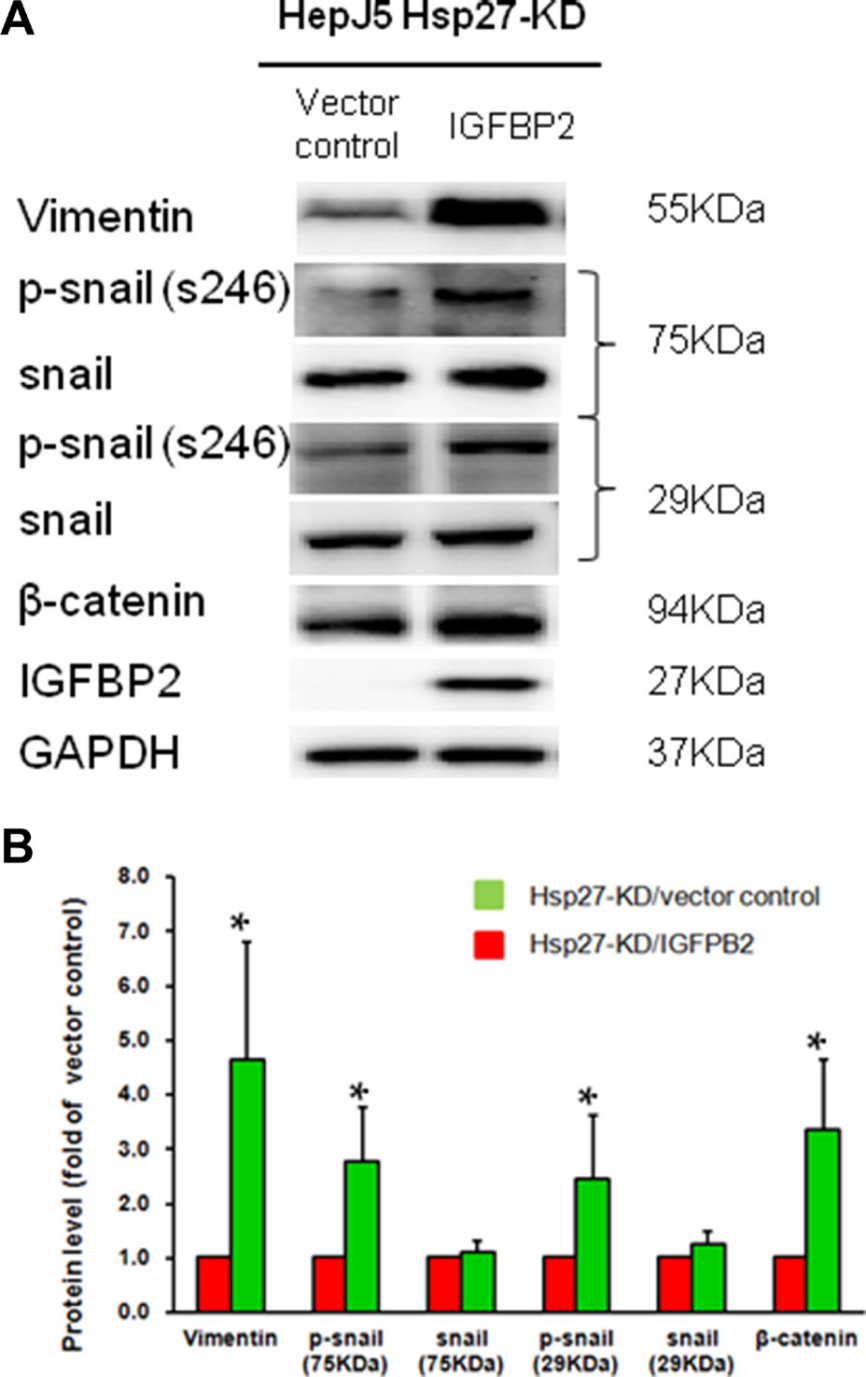
Overexpressing IGFBP2 in HepJ5 Hsp27KD reverses the EMT marker expression pattern (**A**) The EMT expression levels were measured by western blot, and (**B**) quantitative results are shown.

To confirm the role of IGFBP2 in Hsp27-meditated tumor metastasis, we determined the levels of EMT biomarkers in IGFBP2-overexpressing and control Hsp27-KD cells. As shown in Figure 8, the vimentin, β-catenin, and phospho-snail levels were increased in IGFBP2-overexpressing Hsp27-KD cells. These results are consistent with the increased migration and invasion of IGFBP2-overexpressing Hsp27-KD cells. These data demonstrate that Hsp27 mediates tumor metastasis through IGFBP2.

### Hsp27 regulates IGFBP2 expression through indirect gene transcription

**Figure 9:**
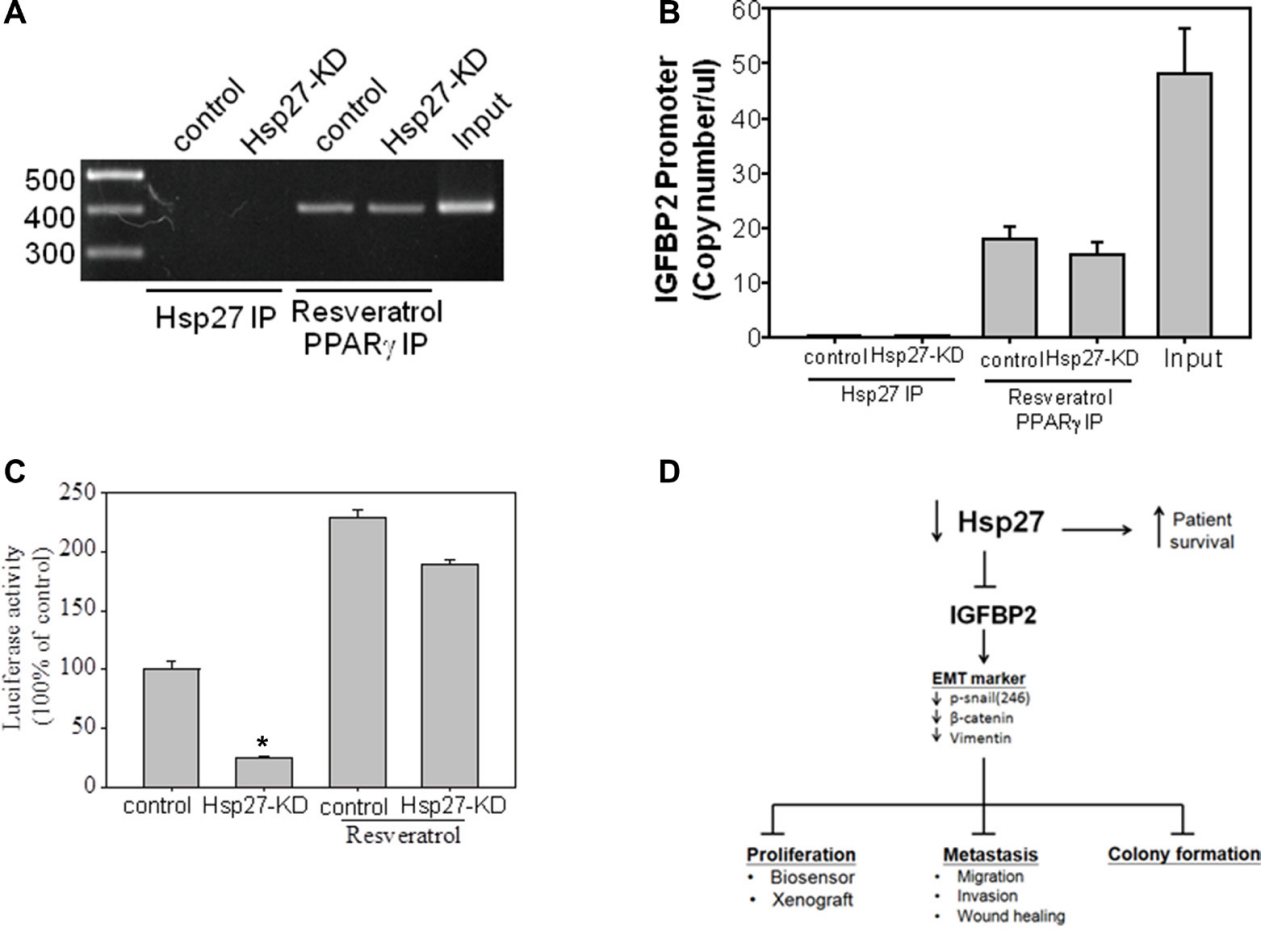
IGFBP2 expression and transcriptional regulation in HCC cells (**A**) IGGBP2 protein expression in five HCC cell lines. (A) A ChIP assay of the IGFBP2 promoter region was performed using the Hsp27 antibody in HepJ5 and HepJ5 Hsp27-KD cells. Resveratrol, which induces PPARγ activation at the IGFBP2 promoter region, was used as a positive control. The IGFBP2 promoter region from −732 to −343 was amplified by PCR from the eluents from each group and analyzed by agarose gel electrophoresis. (**B**) Real-time PCR (Q-PCR) quantitative curve of the eluents from the IGFBP2 ChIP assay. IGFBP2 promoter fragments were measured and compared by Q-PCR analysis. (**C**) The IGFBP2 luciferase assay used the PGL3 vector containing the IGFBP2 promoter region from −1265 to +465. Post IGFBP2-PGL3 transfection on Hep-J5 and Hep-J5 Hsp27KD cells, the luciferase activities were measured and normalized with RLTK activity. The luciferase activity from empty vector was defined as a one-fold. *indicates *p* < 0.05. (**D**) Schematic of Hsp27 regulation in HCC.

To determine whether Hsp27 acts as a transcription factor that directly binds the IGFBP2 promoter, ChIP assays were performed in both scrambled control and Hsp27-KD cells. As a positive control, we used resveratrol to activate the PPARγ protein, which bind the IGFBP2 promoter. As shown in Figure 9A, ChIP assays showed that Hsp27 does not directly bind the IGFBP2 promoter in either scrambled control or Hsp27-KD cells, whereas resveratrol-mediated PPARγ activation was observed at the IGFBP2 promoter region. As shown in Figure 9B, real-time PCR (Q-PCR) assays also showed no gene amplification from eluents of the Hsp27 antibody from the IGFBP2 ChIP assay, whereas eluents from resveratrol-treated HCC cells treated with the PPARγ antibody contained approximately 20 copy numbers of the IGFBP2 fragment per microliter. To confirm that Hsp27 signaling mediates activation of IGFBP2 gene expression at the transcriptional level, we performed an IGFBP2 promoter luciferase assay using scrambled control and Hsp27-KD cells. An approximately 1.7-kb genomic fragment encompassing the human IGFBP2 gene (−1261 to +465) was inserted upstream of a luciferase reporter gene (pGL3 plasmid). Experiments utilizing the full-length construct demonstrated that IGFBP2 promoter activity was significantly reduced (> 4-fold) in Hsp27-KD cells (Figure 9C, **p < 0.05) compared with control HepJ5 cells. Additionally, both scrambled control and Hsp27-KD cells treated with 10 μM resveratrol for 24 h exhibited stronger luciferase activity than cells that were not treated with resveratrol, indicating that positive regulation by resveratrol mediates IGFBP2 expression.

## DISCUSSION

Most heat shock proteins act as chaperones to stabilize new proteins by correcting the folding of or refolding damaged proteins during cell stress. This function also provides cancer cells with the ability to counteract apoptosis signaling and to undergo proliferation [21]. Hsp27 is a major member of the heat shock protein family, and it was found to be correlated with cancer progression and poor prognosis. Hsp27 was recently reported to play an important role as a diagnostic or prognostic marker and to be associated with chemo-resistance in several types of cancer [22]. In pancreatic cancer, the overexpression of Hsp27 increases gemcitabine sensitivity through S-phase arrest and apoptosis [23].

In HCC, Zhang et al. demonstrated that the expression of Hsp27 activates Akt signaling, which promotes MMP2 and ITGA7 expression and HCC metastasis. OGX-427, which targets Hsp27, obviously suppresses HCC metastasis and represents an attractive therapeutic option [24]. Although many studies have indicated that Hsp27 is a potential therapeutic target in HCC treatment, the detailed mechanism through which Hsp27 affects cancer behavior remains unclear. Our study demonstrates that IGFBP2 is a novel downstream gene mediating the growth and invasiveness of HCC. Hsp27 was highly expressed in HepJ5 and Mahlavu cells compared with a normal liver cell line (Figure 1), consistent with clinical results showing a correlation between high Hsp27 expression and poor patient survival (Figure 5). Targeting Hsp27 might influence cell growth, migration and invasion in *in vitro* and *in vivo* systems. In addition, we observed that the silencing of Hsp27 decreased the expression levels of vimentin, phospho-snail, and β-catenin, thus influencing the proliferation and metastasis of HCC cells (Figure 9D). To further dissect the mechanism, we performed a Nanostrip analysis to detect downstream genes of Hsp27 (data not shown), and the results identified IGFBP2 as a possible candidate gene. The Hsp27 levels were correlated with the expression of IGFBP2 in HCC specimens and HCC cell lines (Figure 7B and Supplementary Figure 1). The silencing of Hsp27 caused a decrease in the IGFBP2 levels, resulting in decreased proliferation, metastasis and colony formation *in vitro*. The overexpression of IGFBP2 in Hsp27-KD cells increased the number of migratory and invasive cells (Figure 7D). However, overexpressed-IGFBP2 in Hsp27-KD didn't reverse completely in migration and invasion assay. The possible explain may be the level of IGFBP2 may not reach the amount to achieve the recovery of migratory or invasive ability in Hsp27-KD cells. To further confirm whether Hsp27 directly regulates IGFBP2 expression, we performed ChIP and luciferase assays and found that Hsp27 might indirectly regulate the transcription of IGFBP2 (Figure 9). These data indicate that Hsp27 might mediate progression and metastasis via IGFBP2, thus providing a potential strategy for clinically improving patient survival.

The correlation between cancer and IGFBP2 has seldom been mentioned previously. Some studies showed that the upregulation of IGFBP2 is often associated with aggressiveness and poor prognosis in glioblastoma and breast cancer through regulation of the β-catenin signaling pathway, which is also responsible for tumor invasion, proliferation and the maintenance of glioma stem cells [17, 25–27]. In esophageal cancer, IGFBP2 overexpression is associated with shorter disease-free intervals and treatment resistance, and the knockdown of IGFBP2 sensitizes esophageal adenocarcinoma cells to cisplatin in a serum-dependent manner [28]. The overexpression of IGFBP2 has been reported to be responsible for tamoxifen resistance and recurrence in pre-menopausal women with ERα-positive breast cancer [27]. Inhibiting IGFBP2 improves the sensitivity of bladder cancer cells to cisplatin by elevating the expression of maspin [29]. However, no previous report has defined the relationship between IGFBP2 and HCC.

There are even fewer mentions of the novel correlation between Hsp27 and IGFBP. IGFBP-2 has been demonstrated to be an enhancer of IGF-1 function in several types of cancer [15, 30, 31]. Some studies have noted a relationship between Hsp27 and IGF-1. In castration-resistant prostate cancer, Hsp27 phosphorylation might interact with the IGF-1 signaling pathway and promote cancer progression. Hsp27 knockdown blocks the IGF-1-induced phosphorylation of Erk, p90Rsk, and Akt and enhances cell apoptosis [32]. IGFBP2 is also overexpressed in castration-resistant androgen-independent prostate LNCaP tumors and promotes their proliferation [33]. These studies suggest that Hsp27 and IGFBP-2 are correlated in cancer proliferation and even metastasis.

Bubendorf et al. found that IGFBP2 and Hsp27 are the most consistently overexpressed genes in CWR22R prostate cancer xenografts that are resistant to hormone treatment [34]. Another study found that the overexpression of preferentially expressed antigen of melanoma (PRAME) induces the repression of three genes: Hsp27, S100A4 and p21 [35]. Furthermore, a microarray analysis of PRAME shRNA-silenced leukemic cells revealed that PRAME alters the expression of two additional genes, IL-8 and IGFBP2, that are potentially involved in carcinogenesis and cancer progression.

In conclusion, this study provides the first demonstration of Hsp27 as the upstream regulator of IGFBP2 and indicates that Hsp27 mediates the regulation of HCC cancer metastasis *in vitro* and *in vivo*. We observed that the silencing of Hsp27 decreases the IGFBP2 levels, consequently decreasing tumor proliferation, migration, and invasion, indicating the therapeutic importance of Hsp27 as a target for potentially increasing patient survival.

## MATERIALS AND METHODS

### Chemicals, reagents and cell culture

Triton X-100, Tris-HCl, puromycin, Trypan blue EDTA, ribonuclease-A, and dimethyl sulfoxide (DMSO) were obtained from Sigma Chemical Co. (St. Louis, MO, USA). The anti-snail antibody was purchased from GenScript. The anti-p-snail and anti-Hsp27 antibodies were purchased from Abcam. Antibodies against IGFBP2, β-catenin, vimentin, and GAPDH were purchased from Santa Cruz Biotechnology, Inc. (Santa Cruz, CA). Hep-J5 and Mahlavu cells were gifts from Dr. C. S. Yang, National Taiwan University, and Dr. C. P. Hu, Veterans General Hospital, Taiwan [20, 36, 37]. The HCC cell lines were grown in Dulbecco's modified Eagle's medium (DMEM, Gibco BRL, Grand Island, NY, USA) supplemented with 2 mM L-glutamine, 1.5 g/l sodium bicarbonate, 10% fetal calf serum (FCS; Gibco BRL) and 2% penicillin-streptomycin (10,000 U/ml penicillin and 10 mg/ml streptomycin). The cells were maintained in a 5% CO_2_ humidified incubator at 37°C as previously described [38, 39].

### Knockdown of Hsp27 expression in HCC cells

Hsp27 knockdown (Hsp27-KD) cells were generated using Hsp27-specific short hairpin RNA (shRNA) (National RNAi Core Facility, Academia Sinica, Taiwan) as previously described [20, 38–41]. The target sequence for the human Hsp27 mRNA (NM_001540) was 5′-CCGATGAGACTGCCGCCAAGT-3′. The MISSION non-target shRNA control vector (SHC002) was used as a scrambled control (Sigma Chemical Co.) [38–41]. HCC cells were transfected with the knockdown vector or the parental vector (pLKO.1<-puro) and then selected with puromycin. The transfection and generation of stably transfected cell lines were performed as previously described [40–42].

### Protein extraction and western blot analysis

Target proteins were analyzed by sodium dodecyl sulfate-polyacrylamide gel electrophoresis (SDS-PAGE) and western blotting. Cell lysates were prepared as previously described [38, 39]. The proteins were separated by SDS-PAGE under reducing conditions and electrotransferred onto PVDF membranes (Bio-Rad Laboratories), which were subsequently blotted using anti-target primary antibodies and horseradish peroxidase (HRP)-conjugated secondary antibodies (1:5000). The blots were then visualized with an enhanced chemiluminescence reagent (GE Healthcare, Piscataway, NJ, USA) and imaged with VersaDoc 5000 (Bio-Rad Laboratories) [38, 39].

### Immunofluorescence staining

The cells were seeded on glass coverslips overnight and then fixed in 4% paraformaldehyde for 15 min at room temperature (RT). The fixed cells were permeabilized with 0.1% Triton X-100 and incubated with 5% BSA blocking buffer for 30 min. After washes with PBS, the cells were incubated overnight at 4°C with a rabbit anti-Hsp27 antibody and then for 1 h at RT with CF^TM^ 488-labeled anti-rabbit secondary antibody (Sigma Chemical Co.). The coverslips were then mounted with Vectashield containing DAPI (Vector Laboratories), and the cells were examined by fluorescence microscopy (Olympus America, Inc.).

### Evaluation of proliferation with the x-CELLigence biosensor system

Proliferation and migration were analyzed using an RTCA DP instrument (ACEA Biosciences, Inc., San Diego, CA, USA) as previously described [26, 43]. The cell growth rate was determined in an E-plate 16 (ACEA Biosciences, Inc.). The cells (10,000 per well) were seeded on an E-plate 16 in FCS-containing medium, and the plate was monitored once every 30 s for 4 h and then once every 30 min. The data were analyzed using RTCA software 1.2 (supplied with the instrument).

### Migration assay

Migratory ability was evaluated in a BD Falcon cell culture insert (BD Biosciences) as described previously [26, 43]. Aliquots of 1 × 10^5^ cells suspended in 500 μl of serum-free media were seeded into the upper part of each chamber, and the lower compartments were filled with media containing 10% FCS. After incubation for 24 h, the non-migrating cells were physically removed from the upper surface of the membrane. The cells on the lower surface were stained with 0.1% crystal violet, images of the cells were captured using an Olympus IX71 inverted microscope (Olympus Corp., Tokyo, Japan), and the cells were counted under a microscope at 100× magnification.

### Invasion assay

Cell invasion was evaluated using 24-well BD BioCoat™ Matrigel Invasion Chambers (BD Biosciences). Scrambled control or Hsp27-KD cells (1 × 10^5^) suspended in serum-free medium were seeded into the upper compartments, and the lower compartments were filled with medium containing 10% FCS. After incubation for 18 h, the non-migrating cells were removed from the upper surface of the membrane. The cells on the lower surface were stained with 0.1% crystal violet. Images of the cells were captured using an Olympus IX71 inverted microscope (Olympus Corp., Tokyo, Japan), and the cells were counted using a microscope at 100X magnification [38, 39].

### Wound-healing assay

The wound-healing migration assay was performed using the ibidi culture-insert system [26, 43]. Scrambled control or Hsp27-KD cells (5 × 10^5^ cells) in 70 μl of DMEM containing 10% FCS were seeded into ibidi cell culture inserts (ibidi GmbH, Inc., Munich, Germany). After 24 h, the culture-inserts were removed and placed in media. The cell-free gap was monitored using a time-lapse microscope (Lumascope Model 500× video microscopy). The gap was analyzed with ImageJ software.

### *In vivo* tumor xenograft experiments

All mouse experiments were approved by the Institutional Animal Care and Use Committee (IACUC), Taipei Medical University, and the methods used were in accordance with IACUC-approved guidelines. Five-week-old male Nu/Nu mice were used as the *in vivo* experimental model as described previously [28, 29]. In brief, scrambled control and Hsp27-KD HepJ5 cells were suspended in PBS to a final density of 1 × 10^7^ cells/ml. The cell suspension (0.2 ml) was injected subcutaneously (s.c.) in the bilateral flank of each mouse. The tumor dimensions and body weight were recorded twice per week. The tumor volume was calculated using the equation (L × w^2^)/2, where L and w refer to the larger and smaller dimensions obtained at each measurement, respectively [28, 29]. After four weeks, the mice were sacrificed, and all of the tumors were excised and weighed. Half of the excised tumor tissue was fixed in 10% formalin and embedded in paraffin for immunohistochemical staining, and the other half was snap-frozen in liquid nitrogen for further evaluation.

### Tissue samples and immunohistochemistry

Tissue microarray sets including 80 cases of primary HCC with follow-up information (Catalog Nos. CSA4 and CS4) were purchased from SuperBioChips Laboratories (Seoul, South Korea). The pathological diagnosis and tumor grading of these cases were microscopically reconfirmed by a pathologist. The tissue sections were deparaffinized, rehydrated, and blocked with 3% hydrogen peroxide. Heat-induced antigen retrieval was performed in citric acid buffer (pH 6.0) at 121°C for 10 min using a Decloaking Chamber (Biocare Medical, Concord, CA, USA). The sections were incubated with an Hsp27 antibody (Catalog No. ab2790, 1:750; Abcam PLC, Cambridge, United Kingdom) at 4°C overnight. Hsp27 expression was then detected using the Starr Trek Universal HRP Detection System (Biocare Medical). Appropriate positive and negative controls were included in these assays. The intensity of Hsp27 expression was scored semiquantitatively as weakly positive, moderately positive or strongly positive as previously described [20].

### Chromatin immunoprecipitation (ChIP) assay

ChIP assays of cultured cells were performed as described previously [44]. Briefly, scrambled control and Hsp27-KD HepJ5 cells were seeded in a 10-cm petri dish and treated with 10 μM resveratrol for 6 h. The cells were fixed with a final concentration of 1% formaldehyde directed addition to the cell culture medium at 25°C for 15 min. The crosslinking reaction was stopped by adding 0.125 M glycine for 5 min followed by collection of the cells in a new eppendorf tube. The cell lysate was sonicated three times with 10-s bursts to yield input DNA enriched in fragments of approximately 1,000 bp in size. Hsp27 and PPARγ antibodies were used for the immunoprecipitation reactions. Primers detecting the IGFBP2 promoter region amplified a region from −732 to −343 for 32 cycles (forward: 5′-cacttagggttctcataggttgta-3′ and reverse: 5′-actatgactcctgaaggagaat-3′), and the PCR products were then detected by agarose gel electrophoresis.

### Luciferase activity assay

IGFBP2 promoter-luciferase gene fusions were introduced into the polylinker upstream of the luciferase gene in the vector pGL3-Basic (Promega). The promoter-proximal region was inserted in the KpnI and NheI restriction sites after amplification by PCR with the full-length IGFBP2 sense (−1261; 5′-ggttaactagctgttgaactgaatc-3′) and antisense (+465; 5′-aactacttgttccagaccgcgtta-3′) primers, which anneal to the pGL3-Basic vector downstream of the transcription initiation site. Scrambled control and Hsp27-KD HepJ5 cells were transiently co-transfected with 10 μg of IGFBP2-pGL3 or empty PGL3 plasmid and 1 μg of RLTK plasmid (Promega, Madison, WI, USA) by electroporation. After incubation for 24 h, the medium was changed to culture medium containing 10% FBS. After 24 h, the cells were lysed with 1× Reporter Lysis Buffer (Promega, Madison, WI, USA) and stored at −20°C overnight. Luciferase activity was determined by mixing 50 μl of cell lysate and 50 μl of Luciferase Assay Reagent (Promega). Luciferase light units were read with a HIDEX Chameleon Microplate Reader, and the relative luciferase units were normalized to the Renilla luciferase activity in the same cell lysates. Each luciferase assay was performed three times.

### Electroporation transfection

The Human IGFBP2 overexpression plasmid (RC202573) was purchased from OriGene Technologies, Inc. (Rockville, MD, USA). The transfection protocol has been described previously [45]. Briefly, 5 × 10^6^ cells were washed twice with phosphate-buffered saline and mixed with 10 μg of plasmid. One pulse was applied for a duration of 20 ms under a fixed voltage of 1.2 kV on a pipette-type microporator MP-100 (Digital Bio, Seoul, Korea).

### Statistical analysis

The associations between clinicopathological characteristics and Hsp27 expression in HCC were analyzed using the Chi-square test. An overall survival curve was generated using the Kaplan-Meier method and evaluated by the Log-rank test. *p* < 0.05 was considered statistically significant. All statistical calculations were performed using SPSS statistics 17.0 software (SPSS, Inc., Chicago, IL, USA).

## SUPPLEMENTARY MATERIALS FIGURE



## Abbreviations

Hsp27: heat shock protein 27
HCC: hepatocellular carcinoma.
