# Supplementary material for: IGFBP2 plays an important role in heat shock protein 27-mediated cancer progression and metastasis
Source: Oncotarget. 2017 Jul 5;8(33):54978–92. doi: 10.18632/oncotarget.18989 (PMC5589635; doi:10.18632/oncotarget.18989)
Supplement: Supplementary file 1 [file oncotarget-08-54978-s001.pdf]

IGFBP2 plays an important role in heat shock protein 27-mediated cancer progression and metastasis

SUPPLEMENTARY MATERIALS

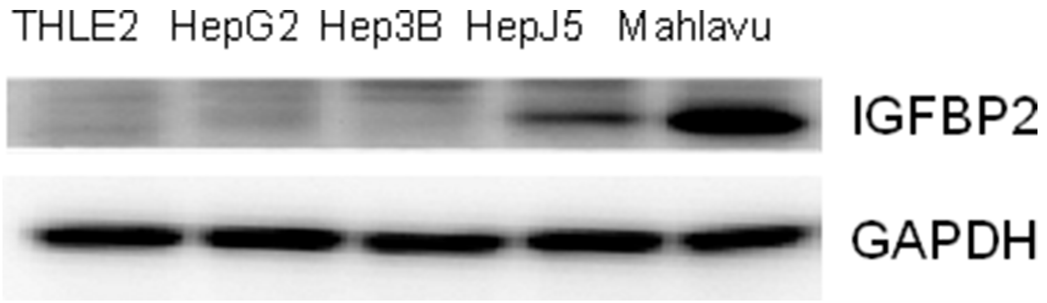

Supplementary Figure 1: IGFBP2 expression in HCC cells. IGGBP2 protein expression was determined by western blotting in normal liver cell line and HCC cell lines. GAPDH was used as an internal control.
